# Human milk oligosaccharides are distinctly processed by early life gut microbial sialidases

**DOI:** 10.1080/19490976.2026.2748788

**Published:** 2026-10-29

**Authors:** Olivia L. Plisko, Valen S. Chapel, Josh J. Sekela, Julie A. Talbert, Steven D. Townsend, Babette S. Zemel, Gary D. Wu, Matthew R Redinbo

**Affiliations:** a Department of Biochemistry and Biophysics, University of North Carolina, Chapel Hill, NC, USA; b Department of Chemistry, University of North Carolina, Chapel Hill, NC, USA; c Department of Chemistry, Vanderbilt University, Nashville, TN, USA; d School of Medicine, University of Pennsylvania, Philadelphia, PA, USA; e Department of Microbiology and Immunology, and Integrative Program for Biological and Genome Sciences, University of North Carolina, Chapel Hill, NC, USA

**Keywords:** Sialic acid, sialidases, human milk oligosaccharides, infant gut microbiome, infant feeding method

## Abstract

The infant gut microbiome in early life is shaped by numerous factors, including feeding method. Exclusively breastfed and formula-fed infants exhibit distinct microbiome compositions. While these effects are well-established, the underlying mechanisms that drive these differences are not fully understood. Here, we examine the removal of sialic acid from human milk oligosaccharides (HMOs) influenced by human microbial sialidases in early life. We identify 351 and 214 unique sialidase sequences from adult and infant microbiome metagenomes, respectively, and characterize a diverse, representative set *in vitro* using sialylated HMOs normally present in human breastmilk. We find these proteins exhibit distinct abilities to metabolize 3’- and 6’-sialyllactose, which are the most abundant sialylated HMOs in breastmilk, and distinctly process other more complex substrates, as well. We provide structural insights into these functional differences by pinpointing active site features uniquely present in efficient enzymes and show that these results extend to activities on complex mixtures of sialylated HMOs purified from human breastmilk. Finally, we demonstrate in human clinical data that distinct profiles of sialidase-encoding bacterial taxa are observed in breastfed and formula-fed infants at 1 and 4 months of age, prior to the introduction of solid foods. Together, these findings show that specific bacterial sialidases are influential in shaping the early gut microbiome, data that may improve the supplementation of infant formulas for enhanced developmental outcomes.

## Graphical abstract

**Figure:**
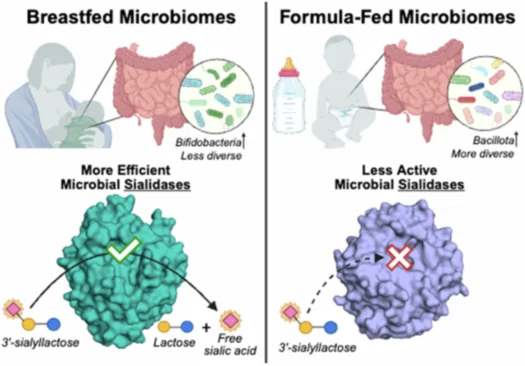

## Introduction

Early life is a critical time for the establishment and development of the gut microbiome.^1^ Several factors influence microbiome compositions during the days, weeks, and even months following birth, including mode of delivery, gestational age, and maternal and infant antibiotic exposure.^2^ Primary feeding also plays a crucial role—exclusively breastfed and exclusively formula-fed infants have markedly distinct gut microbial compositions during the first six months of life, prior to the typical introduction of other nutrient sources. Breastfed infant microbiomes have low taxonomic diversity; however, the bacteria present tend to have more beneficial properties, including high abundances of *Bifidobacterium*.^3-5^ Conversely, formula-fed infants have higher taxonomic diversity, but this creates opportunities for the growth of less beneficial or even pathogenic taxa. More broadly, breastfed infants have been reported to have numerous other health advantages, such as reduced risk of necrotizing enterocolitis, increased cognitive function, improved immunological development, decreased atopy, and lower risk for obesity and Type II diabetes later in life.^2-4^ ^,^ ^6-8^

Breastmilk and formula ostensibly provide all essential nutrients for the growth and development of the infant. While the production of infant formula is highly regulated to meet these strict nutritional standards, there are still significant compositional differences compared to breastmilk.^9^ Notably, total oligosaccharide concentrations are significantly distinct: 5–15 g/L in mature human milk compared to 0.05 g/L in mature bovine milk, after which infant formulas are modeled.^9-12^ More specifically, the concentrations of sialylated milk oligosaccharides differ between the two nutrient sources. Sialic acid, more specifically *N*-acetylneuraminic acid (Neu5Ac), is an acidic nine-carbon sugar that is often appended via α2-3 or α2-6 linkages to terminal galactose or *N*-acetylglucosamine moieties in milk oligosaccharides.^11^ ^,^ ^13^ About 10%–20% of human milk oligosaccharides (HMOs) in breastmilk are sialylated, in typical concentrations from 2 to 3 g/L in mature milk.^10^ ^,^ ^14^ Alternatively, bovine milk contains approximately 100-fold lower concentrations of sialylated milk oligosaccharides, reaching only ~0.04 g/L.^13^ ^,^ ^15^ While few studies have characterized the concentrations of sialylated milk oligosaccharides in commercial infant formulas, one reported 0.05–0.10 g/L in nine bovine milk-based formulas intended for full-term infants under 6 months of age.^15^ They also observed that 70% of the total sialic acid was bound to glycoproteins; however, in breastmilk 70%–80% of the total sialic acid is conjugated to HMOs, revealing distinct sialic acid profiles in formula and breastmilk.^13^ ^,^ ^15^ ^,^ ^16^

Sialylated HMOs remain undigested through the stomach and small intestine, as human sialidases are not present in these locations.^17-19^ However, gut microbes are known to produce sialidases that catalyze sialic acid removal (Figure 1A).^20^ ^,^ ^21^ Some bacteria utilize additional machinery to further metabolize sialic acid as a carbon source, often producing beneficial short-chain fatty acids (SCFA) as a byproduct.^20^ ^,^ ^22^ However, sialic acid can also be absorbed by the intestines and reach host tissue via the circulation.^18^ Sialic acid obtained by the diet is highly beneficial to infants, playing important roles in neurodevelopment and cognitive function, intestinal maturation, immunomodulation, and resistance to respiratory and intestinal pathogens.^12^ ^,^ ^18^ ^,^ ^23^

**Figure 1.**
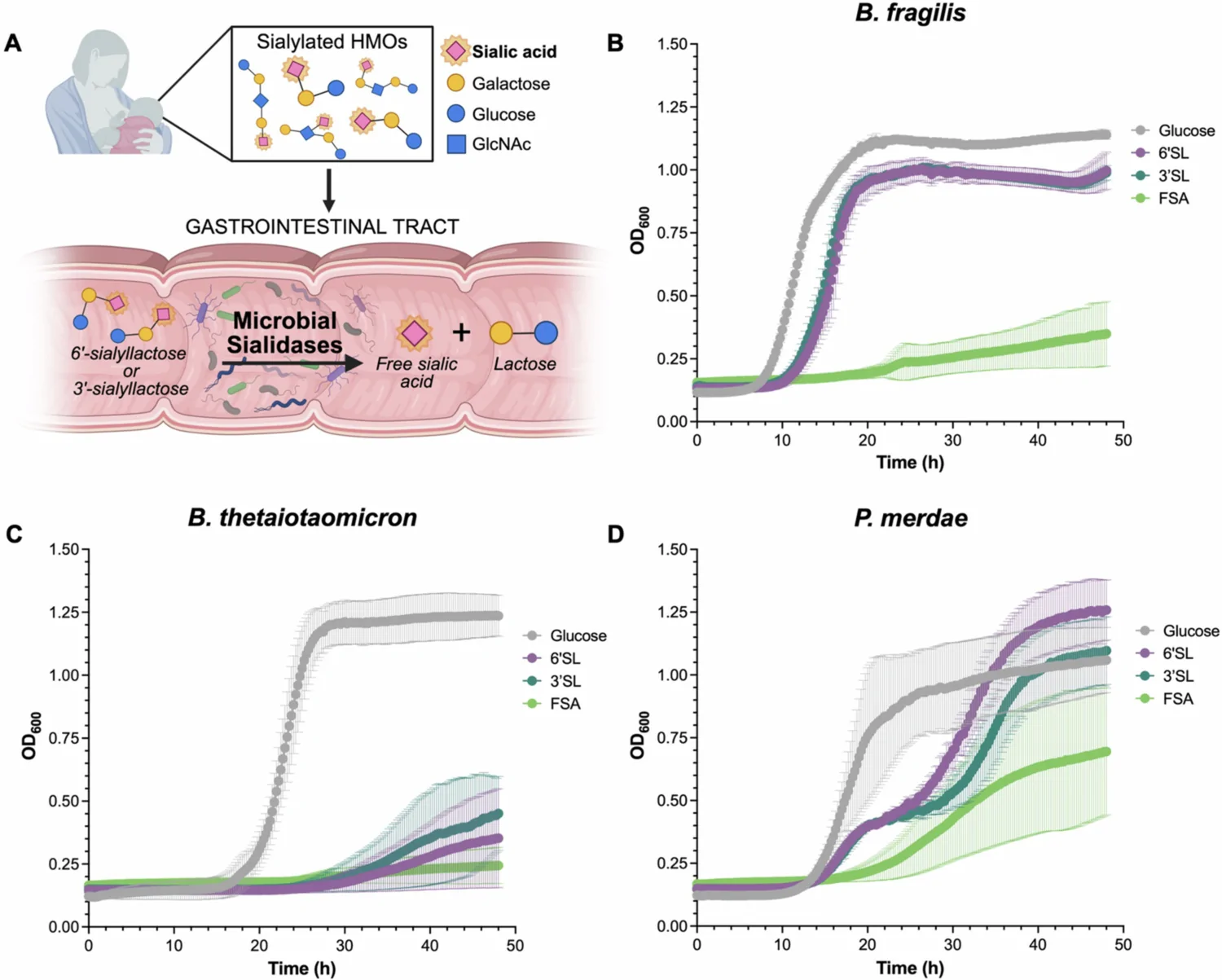
Distinct utilization of sialic acid-containing compounds by gut commensal microbes. (A) Schematic overview of sialylated human milk oligosaccharide (HMO) utilization by the infant gut microbiome. Growth curves of *B. fragilis* (B), *B. thetaiotaomicron* (C), and *P. merdae* (D) with 1% w/v of the following as sole carbon sources: glucose, free sialic acid (FSA), and the human milk oligosaccharide components 3’-sialyllactose (3’SL) or 6’-sialyllactose (6’SL). Error bars represent standard error of the mean (SEM) of *N* = 3 biological replicates.

Microbial sialidases have been broadly characterized in terms of overall structure and catalytic mechanism. The majority of bacterial sialidases are classified in the glycoside hydrolase family 33 (GH33) that adopt a conserved 6-bladed *β*-propeller fold.^24^ GH33 sialidases employ a conserved catalytic mechanism using a tyrosine nucleophile activated by an adjacent glutamic acid. A covalent intermediate is formed between the tyrosine hydroxyl and sialic acid.^21^ ^,^ ^25^ ^,^ ^26^ This link is broken by a water molecule in the case of hydrolytic sialidases or by the O7-hydroxy group of the sialic acid for intramolecular *trans*-sialidases.^20^ ^,^ ^27^ ^,^ ^28^ While some microbial sialidases have been characterized for *in vitro* sialylated HMO processing ability, the differences in assay conditions and readout makes it difficult to compare functionality with across species.^29-35^ Despite the importance of sialic acid to infant development, and the fact that host factors do not participate in obtaining it from diet, there is a relative dearth of knowledge about the functions of gut microbial sialidases in early life. Additionally, while the connections between HMO abundance and microbiome composition have been established, the molecular mechanisms driving these differences remain unclear.

Here, using a combination of bacterial culturing, metagenomic analyzes, *in vitro* enzyme activity assays, and protein crystallography, we map the diversity of microbial sialidases in the adult and infant gut microbiomes and examine their distinct abilities to metabolize sialylated HMOs. We find that individual sialidase enzymes can uniquely process structurally diverse HMOs and we identify specific structural moieties that define efficient sialidase function. We also demonstrate that common gut microbes variably utilize sialylated HMOs for growth and show that breastfed and formula-fed infants exhibit significantly different sialidase-encoding microbes in early life. Taken together, our results support the conclusion that the ability of gut microbial sialidases to metabolize sialylated HMOs plays an important role in shaping the infant gut microbiome. These insights provide a molecular mechanism that helps to explain the differences in microbial compositions observed between breastfed and formula-fed infants.

## Materials and methods

### Anaerobic culturing

Type strains for *Bacteroides fragilis* (ATCC 25285), *Bacteroides thetaiotaomicron* (ATCC 29148), and *Parabacteroides merdae* (ATCC 43184) were purchased from American Type Culture Collection (ATCC) as glycerol stocks for *B. fragilis* and *B. thetaiotaomicron*, and freeze-dried powder for *P. merdae*. The type strain of *Bifidobacterium longum* subsp. *infantis* (ATCC 15697) was obtained as a glycerol stock from the UNC Microbiome Core's AMC Culture Collection. Glycerol stocks were stored at −80 °C and freeze-dried powder was stored at 4 °C until use.

*B. fragilis*, *B. thetaiotaomicron*, and *P. merdae* were grown in yeast, casitone, fatty acid (YCFA) media. Glycerol stocks were activated by decanting into 10 mL aliquots of YCFA media. Freeze-dried powder was activated by resuspending in 1 mL YCFA, then decanting into a 10 mL aliquot of YCFA. YCFA media was supplemented with 100X vitamin solution (ATCC) immediately before inoculation. Bacteria were subcultured twice prior to growth assays. Overnight 10 mL cultures were grown for 48 hours, prior to harvesting cells from 1 mL aliquots via centrifugation. Pelleted cells were washed three times with phosphate buffered saline (PBS), then final pellets were resuspended in 1 mL fresh PBS. Resuspended bacteria were diluted 1:100 in YCFA containing 1% w/v carbon source (glucose, 6’-sialyllactose (6’SL), 3’-sialyllactose (3’SL), or free sialic acid (FSA). 200 μL of inoculated culture was plated into 96-well flat-bottom cell culture plates (Costar). Changes in OD_600_ were monitored using a BioTeck Epoch2 microplate spectrophotometer (Vermont) inside the anaerobic chamber, taking readings every 15 minutes for 48 hours.

Cultures for *B. longum* subsp. *infantis* were grown in De Man, Rogosa, and Sharpe (MRS) media (Alpha Biosciences) supplemented with 0.05% w/v L-cysteine. Overnight 10 mL cultures were supplemented with 1% w/v glucose and 1% w/v stachyose to improve cell viability. Bacteria were subcultured twice prior to growth assays. Overnight cultures were grown for 24 hours, then 1 mL aliquots were harvested via centrifugation, and washed three times with MRS media devoid of carbon source. Final pellets were resuspended in 1 mL MRS media devoid of carbon source. Resuspended bacteria were diluted 1:100 in MRS media containing 2% w/v carbon source (glucose, lactose, 6’SL, 3’SL, or FSA). Inoculated cultures were plated and monitored as described above.

### Sialidase identification and SSN creation

Sequences of bacterial sialidases were identified using a two-step structure-based approach akin to that used previously for gut microbial enzymes.^36-40^ Seven previously solved structures of microbial sialidases were selected as reference sequences: *B. thetaiotaomicron* (4BBW),^29^ *Streptococcus pneumoniae* (5F9T),^41^ *Clostridium perfringens* (2BF6),^42^ *Alistipes sp.* (6MNJ),^43^ *Micromonospora virifaciens* (1EUR),^44^ *Cutibacterium acnes* (7LBU),^45^ and *Ruminococcus gnavus* (4X47).^46^ First, all 9,879,896 protein sequences from the Integrated Gene Catalog (IGC) of metagenomes assembled from 1267 adult human fecal samples were aligned to the reference sequences using Protein-Protein BLAST.^47^ Sequences with 25% sequence similarity or higher to at least one reference sequence were advanced to the next step. This second step ensured that each chosen sequence contained the 5 conserved active site residues essential for sialidase activity (Figure 2B). In total, 395 sequences were identified that met both criteria. That set was further condensed for redundancy with a 95% similarity threshold using the Enzyme Function Initiative-Enzyme Similarity Tool (EFI-EST), producing a list of 351 unique gut microbial sialidase sequences.^48^ ^,^ ^49^ The resultant proteins were then organized based on sequence similarity using EFI-EST and visualized as a sequence similarity network using Cytoscape (v.3.10.1). Taxonomy labels were assigned using BLASTP, and sequences were renamed accordingly.

**Figure 2.**
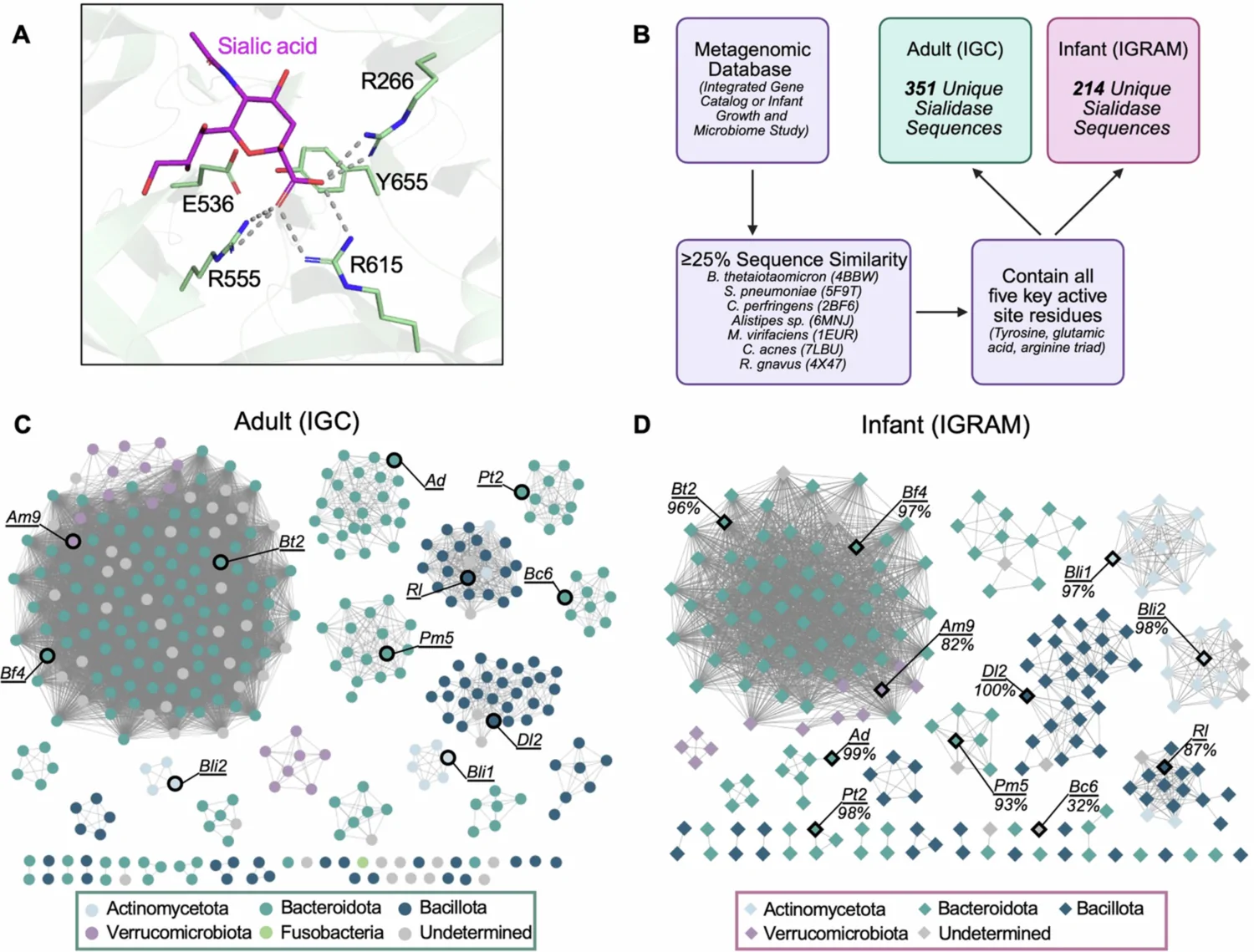
Diverse gut microbial sialidases in adult and infant microbiomes. (A) Conserved active site residues of *C. perfringens* sialidase co-crystallized with free sialic acid (2BF6), with arginine triad polar contacts highlighted. (B) Metagenomic pipeline and criteria used to identify gut microbial sialidases. (C) Sequence similarity network (SSN) of the 351 unique microbial sialidases identified in the adult gut microbiome from the Integrated Gene Catalog (IGC) database of fecal metagenomics (E = 1 × 10^−90^). Sequences chosen for further characterization are bolded and labeled. (D) SSN of the 214 unique microbial sialidase sequences identified in the infant gut microbiome from the Infant Growth and Microbiome Study (IGRAM) database of infant fecal metagenomics (E = 1 × 10^−90^). The closest counterparts to the selected IGC sequences are bolded, with percent identities indicated.

A similar workflow was used to identify microbial sialidases in the infant gut microbiome using the Infant Growth and Microbiome study (IGRAM), consisting of 153 healthy, full-term infants, with samples collected from 4 d to 24 months after birth.^50-52^ All sequencing data used in this analysis was previously uploaded to NCBI Sequence Read Archive (accension PRJNA1042647).^52^ A total of 214 unique microbial sialidase sequences were identified from IGRAM. A multiple sequence alignment was performed using Clustal Omega (v.1.2.4) for all sequences identified from both IGRAM and the sequences chosen from the IGC for recombinant expression and purification. The closest match in IGRAM to each IGC sequence was identified, then a pairwise sequence alignment was performed for each pair using EMBOSS Needle to calculate sequence identity.

### Protein expression

Selected sialidase sequences were assessed for signal peptides using SignalP 6.0.^53^ Predicted signal peptides were removed from the sequences to optimize protein expression. Additionally, a large C-terminal domain was removed from the *Rl* sequence, and large *N*-terminal and C-terminal domains were removed from the *Pt2* sequence for improved purification of the catalytic domains. Multiple sequence alignments and AlphaFold 2 models were used to determine the location of the catalytic domains to make appropriate truncations of the additional *N*- and C-terminal domains. Genes for sialidase sequences chosen for recombinant expression were codon-optimized for *E. coli* and commercially synthesized. They were ligated into a pLIC-His vector and purchased from Bio Basic, except for *Bli2*, which was ligated into a pET28(a) vector and purchased from Twist Bioscience. Plasmids were transformed into BL−21 (DE3) *E. coli*, and overnight cultures were grown in 100 mL of lysogeny broth (LB) with 100 μg/mL ampicillin or 50 μg/mL kanamycin for pLIC-His and pET28(a), respectively. Saturated overnight cultures were used to inoculate 1.5 L terrific broth (TB) containing the same concentration of the appropriate antibiotic as the overnight culture and ~2 μL of Antifoam 204. TB cultures were shaken at 200 rpm and 37 °C until reaching an OD_600_ of ~1.0. Protein expression was induced by adding 100 mM isopropyl beta-D-1-thiocalactopyranoside (IPTG); the temperature was dropped to 18 °C, and cultures were allowed to shake at 200  rpm overnight. Cells were pelleted the next day by centrifugation at 4200 × g at 4 °C in a Sorvall RC-3B Refrigerated Centrifuge. After media removal, cell pellets were flash-frozen using liquid nitrogen and stored at −80 °C until protein purification.

### Protein purification

Thawed cell pellets were resuspended in 35 mL NiA buffer (25 mM HEPES, 500 mM NaCl, 50 mM Imidazole, pH 7.5) with DNase, complete-EDTA-free protease inhibitor tablet (Roche), and lysozyme. Cells were lysed via sonication, then centrifuged at 17,000 rpm at 4 °C for 1 hour in an Avanti J-E centrifuge to pellet cell debris. Cell lysates were filtered using 0.22 μm PES filter. Filtered lysates were flowed over a HisTrap FF column (Cytiva), then loaded onto the Akta pure FPLC system. The column was washed with NiA buffer, and protein was eluted with NiB buffer (25 mM HEPES, 500 mM NaCl, 250 mM Imidazole, pH 7.5). Fractions containing eluted protein according to UV detection were pooled and injected onto a HiLoad 16/600 Superdex 200 size exclusion column (Cytiva). Proteins were eluted from the size exclusion column using S200 buffer (*Bt2* and *Bf4*: 25  mM HEPES, 50 mM NaCl, pH 7.5; all other proteins: 25 mM HEPES, 300 mM NaCl, 5% glycerol, pH 7.5). Protein containing fractions were pooled and purity was confirmed via SDS-PAGE. Pure samples were concentrated using an mPulse (Formulatrix). Samples were aliquoted, snap-frozen in liquid nitrogen, and stored at −80 °C until further use.

### Reporter substrate sialidase assays using 4MU-Neu5Ac

Sialidase activity was first assessed using the reporter substrate 4-methylumbeliferone-*α*-*N*-acetylneuraminic acid (4MU-Neu5Ac; Neu5Ac is synonymous with sialic acid). Continuous fluorescence-based assays were conducted in 96-well half-area black polystyrene plates (Costar) with a final reaction volume of 100 μL. A pH screen was conducted for each enzyme using an optimized concentration of sialidase, and pH screen buffer (25 mM NaCl, 20 mM sodium phosphate, 100 mM citric acid) ranging from pH 3–8. Subsequent specific activity assays were conducted using the same optimized sialidase concentration, and assay buffer (15 mM Tris:HCl, 25 mM NaCl) at pH 6.5, which was adequate for all enzymes. Reaction plates were incubated at 37 °C for 5 minutes without 4MU-Neu5Ac, and five initial fluorescence readings (Excitation: 320 nm; Emission: 445 nm) were taken to establish a baseline. The reactions were initiated by adding 50  μM 4MU-Neu5Ac, then the production of 4MU was monitored by measuring the increase in fluorescence over time at 37 °C using a CLARIOstar Plus Microplate Reader. Measured fluorescence values were converted to concentration of 4MU using a standard curve. Initial velocity was determined by completing an iterative linear fit of the change in 4MU concentration over time, then divided by enzyme concentration to calculate specific activity.

### HMO sialidase assays

Pure sialylated HMOs (3’SL and 6’SL: Caymen Chemicals; LSTa, LSTb, LSTc: Dextra) were resuspended in 100% DMSO at 5 mM. Endpoint assays were composed of 100  μM sialylated HMO, assay buffer, and an optimized concentration of pure sialidase. Plates were incubated at 37 °C for 5 minutes prior to initiation of the reaction by the addition of sialylated HMO. Reactions were quenched using 30 μL of 100% LC/MS grade acetonitrile at 6 timepoints ranging from 5 to 30 minutes. Plates were sealed at 37 °C between timepoints. Sialidases that were determined to have no activity were further examined with 1-hour endpoint assays instead of initial velocity timepoints. Once all reactions were quenched, 100 μM *N*-acetyl-D-[1,2,3-^13^C_3_] neuraminic acid (Omicron Biochemicals) was added to each well as an internal standard. Samples were transferred to 1.7 mL tubes and centrifuged at 13,000 rpm for 10 minutes at 4 °C using a Prism R Refrigerated Microcentrifuge (Labnet). In total, 50  μL of each sample was transferred to an autosampler vial and stored at −20 °C until LC/MS-MS analysis.

Endpoint assays using a mixture of sialylated HMOs (sHMO) from breastmilk were conducted using the same methods as the HMO-specific activity assays. Assays were composed of 0.5  mg/mL sHMO diluted in 100% water, assay buffer, and 20 nM sialidase. Reactions were quenched after 1 hour using 30 μL 100% LC/MS-grade acetonitrile. Samples were analyzed via LC/MS-MS immediately after final sample preparation was complete.

Concentration of FSA in each sample was determined via liquid chromatography coupled tandem mass spectrometry (LC/MS-MS). Specific activity data for *Ad*, *Am9*, *Bf4*, *Bt2*, *Pm5*, *Dl2*, *Rl*, *Pt2*, and *Bc6* were collected using an Agilent 1260 Infinity II liquid chromatography system. Specific activity data for *Bli1*, *Bli2*, and *Ad* W361A, and sHMO mixture turnover assays were collected using an Agilent 1290 Infinity II liquid chromatography system. Activity data for *Pm5* W169F were collected using a Waters Acquity Classic UPLC system. Sample injection volume was 5 μL for both Agilent systems, and 10 μL for the Waters system. The autosampler temperature was kept at 8 °C. Separation conditions were the same for every instrument and used an InfinityLab Poroshell 120 EC-C18 column (4.6 × 150 mm, 2.7 mm) at 40 °C. Solvent A was 100% H_2_O and 10 mM ammonium carbonate and Solvent B was 80% LC/MS grade acetonitrile, 20% H_2_O, and 10 mM ammonium carbonate. Samples were eluted isocratically with a flow rate of 0.3  mL/min with 87.5% Solvent A and 12.5% Solvent B. Sialic acid retention time was roughly 4.4 minutes.

An Agilent 6460 A QQQ LC/MS was employed for all specific activity data collected for *Ad*, *Am9*, *Bf4*, *Bt2*, *Pm5*, *Dl2*, *Rl*, *Pt2*, and *Bc6*. Specific activity data for *Bli1*, *Bli2*, and *Ad* W361A, and sHMO mixture turnover assays were collected using an Agilent 6475 LC/TQ. Lastly, *Pm5* W169F activity was quantitated using a ThermoScientific TSQ Vantage Ultra Triple Quad mass spectrometer. Ionization conditions were the same for all three instruments. Ionization was monitored using electrospray ionization in negative mode. For sialic acid, the transition at 308 → 87 *m/z* was monitored. For C_13_-labeled sialic acid, the transition at 311 → 90 *m/z* was monitored.

### Protein crystallography

Crystals for *Ad* and *Pm5* were grown using sitting-drop vapor diffusion, while crystals for *Rl* were grown via hanging-drop vapor diffusion. All crystals were grown at 20 °C. Sitting-drop trays were set using an NT8 crystallization robot (Formulatrix) with Hampton Research 3-well midi crystallization plates (Swissci). Hanging drop trays were set manually using EasyXtal 15-well tool trays (Qiagen). The specific crystallization conditions are as follow. *Ad*: 0.1 M Sodium Citrate:HCl pH 4, 0.8 M ammonium sulfate. Crystals formed in a 1:2 protein (15 mg/mL) to crystallant. *Ad* was kept in S200 purification buffer and incubated with 1 mM FSA in water prior to setting the drops. *Pm5*: 0.2 M NaCl, 0.1 M Tris:HCl pH 8.5, 25% PEG 3350. Crystals formed in 1:1 protein (15.6 mg/mL) to crystallant. *Pm5* was buffer exchanged into crystallography buffer (25  mM HEPES, 50  mM NaCl, pH 7.5) and incubated with 1 mM *N*-acetyl-2,3-dehydro-2-deoxyneuraminic acid (DANA) in 100% DMSO prior to setting drops, but no density was observed for DANA in the resultant electron density maps. *Rl*: 0.2 M ammonium sulfate, 0.1 M Bis-Tris pH 5.5, 27.5% PEG 3350. Crystals formed in a 1:1 protein (16 mg/mL) to crystallant ratio. *Rl* was buffer exchanged into crystallography buffer and incubated with 1 mM FSA in 100% DMSO prior to setting the drops, but no density for sialic acid was observed in the resultant electron density maps.

Crystals for *Pm5* were cryoprotected in 20% ethylene glycol in crystallant. *Ad* and *Rl* crystals were cryoprotected in 20% glycerol in crystallant. Crystal specimens were then looped and flash cooled in liquid nitrogen. X-ray diffraction data were collected at Brookhaven National Labs National Synchrotron Lightsource II using 17-ID-1 *(Ad*) and 17-ID-2 (*Pm5* and *Rl*). Datasets were processed using AutoPROC with further scaling in CCP4 (v9.0.009) for *Pm5* and *Rl*. AlphaFold models for each enzyme were generated using ColabFold (v1.5.5) and processed using Phenix. Molecular replacement was completed using Phenix (v1.21-5207) with AlphaFold models as search models. Models were refined using Autobuild and phenix.refine, as well as manual adjustments in Coot (v0.9.8.96). Final structures were visualized using PyMOL (v3.1.5.1). Structures were deposited to the Protein Data Bank under the following codes: *Ad*, 10EI; *Pm5*, 10EM; *Rl*, 10EW (Table S1).

### Modeling of 3’-sialyllactose

The structure of 3’-sialyllactose (3’SL) from the cocrystal structure of *Trypanosoma cruzi* trans-sialidase (1S0I) was used to model 3’SL binding to *Ad*. The sialic acid portion of 3’SL was overlayed using PyMOL with the sialic acid monosaccharide co-crystalized in the structure of *Ad*. The glycosidic bonds were then manually rotated in PyMOL to minimize clashes with amino acid sidechains.

### Site-directed mutagenesis

The *Ad* W361A and *Pm5* W169F mutants were created using site-directed mutagenesis with Phusion DNA polymerase (New England Biolabs). Primers were designed using the Agilent QuikChange Primer Design tool (https://www.agilent.com/store/primerDesignProgram.jsp;). Primers were synthesized by Integrated DNA Technologies (Table S2). The mutations were confirmed via sequencing, then transformed into BL21 (DE3) *E. coli* (New England Biolabs). The mutants were expressed and purified as described for wild-type *Ad* and *Pm5*. Activity was assessed using 4MU-Neu5Ac, 3’SL, and 6’SL as described above.

### Circular dichroism

The secondary structures of the *Ad* wildtype, *Ad* W361A, *Pm5* wildtype, and *Pm5* W169F proteins were confirmed using circular dichroism (CD). Protein samples were buffer exchanged into CD buffer (10 mM potassium monophosphate, 100 mM potassium fluoride, pH 7.5) and diluted to 0.2 mg/mL for both *Ad* enzymes, 0.15 mg/mL for *Pm5* wildtype, and 0.1 mg/mL for *Pm5* W169F. Spectral scans were collected from 185 to 260 nm at 25 °C in a 1 mm cuvette using a Chirascan Plus-Spectropolarimeter. A blank scan was collected and subtracted from the sample scans to account for background signal. Collected CD spectra were converted to mean residue ellipticity (MRE) using the below equation where θ is MRE, signal is CD signal (millidegrees), MW is protein molecular weight (g/mol), l is path length (cm), c is protein concentration (g/L), and r is the number of residues in the protein.

$$\theta =\frac{\mathrm{signal}\;*\;\mathrm{MW}}{10\;*\;l\;*\;c\;*\;r}$$

Melting curves were also collected from by monitoring molar ellipticity at 215 nm from 25 to 95 °C for all 4 proteins. Collected melting curves were converted to fraction folded (*α*) using the below equation where *α* is fraction folded, θ is MRE, $\theta_{F}$ is MRE for fully folded protein, and $\theta_{D}$ is MRE for fully denatured protein.

$$\alpha =\frac{\theta -\theta_{D}}{\theta_{F}-\theta_{D}}$$

### Nano-differential scanning fluorimetry

Nano-differential scanning fluorimetry (nanoDSF) was employed to confirm melting profiles observed via CD for *Pm5* wild-type and *Pm5* W169F. Samples that were buffer-exchanged and diluted as described above for CD, were loaded into Prometheus NT.48 Capillaries. Thermal denaturation experiments were conducted using a Nanotemper Prometheus NT.48 system, monitoring intrinsic tryptophan fluorescence at 330 and 350 nm over the range of 25–95 °C increasing at 1 °C per minute.

### Human milk oligosaccharide isolation

Human breast milk was obtained from a single lactating female between 3 d and 3 months postpartum and stored at −20 °C. The deidentified milk sample was provided by Dr. J. Hendrik Weitkamp from the Vanderbilt University Medical Center Department of Pediatrics under a collection protocol approved by the Vanderbilt University Institutional Review Board (IRB #100897). Milk samples were thawed and centrifuged at 3750 rpm for 45 minutes. Subsequently, lipid components were removed by skimming. The proteins were removed by diluting with 1:1 v/v absolute ethanol at 4 °C and centrifuging at 3750 rpm for 45 minutes. The HMO-containing supernatant was concentrated in vacuo, dissolved in phosphate buffer (pH 6.5, 0.2 M), and heated to 37 °C. *β*-galactosidase from *Kluyveromyces lactis* was added, and the reaction mixture was stirred until deemed complete by thin-layer chromatography (TLC). The reaction mixture was diluted with ethanol at 4 °C, centrifuged, and concentrated *in vacuo*. The remaining glucose and galactose were separated from the HMOs using size exclusion chromatography with P-2 Gel (H_2_O eluent). The HMOs were dried by lyophilization.

### Anion-exchange chromatography fractionation of HMOs

Human milk oligosaccharides (HMOs; 500 mg per run) were fractionated using an anion-exchange chromatography protocol adapted for QAE Sephadex A-25 (GE Healthcare/Cytiva). Approximately 14 g of dry resin was hydrated according to the manufacturer's instructions to yield ~100 mL of swollen resin, which was packed into a 2.5 cm internal diameter glass column to a bed height of ~20 cm (total bed volume ~100 mL). Two Tris-based (2 mM) elution buffers were prepared at 0 and 20 mM NaCl concentrations yielding buffers A and B, respectively. Each buffer was adjusted to pH 7.5–8.0 using HCl or NaOH as needed. The column was equilibrated with 5 column volumes (CV; ~500 mL) of buffer A. HMOs were dissolved in 10 mL of buffer A, clarified by filtration through a 0.22 μm syringe filter, and applied to the equilibrated column under gravity flow. Stepwise elution was performed using 500 mL of each buffer. Buffer A was used to collect the neutral/fucosylated HMOs and buffer B was used to collect the sialylated HMOs. Each HMO-containing fraction was concentrated by rotary evaporation and resuspended in approximately 10 mL of water. Desalting was performed by size exclusion chromatography on a P2 gel filtration column (Bio-Gel P2, Bio-Rad). Fractions containing HMOs without detectable salt were pooled. Final HMO fractions were lyophilized and stored at –20 °C until further analysis.

### Mass spectrometry analysis of HMOs

Dried HMO samples were reconstituted in water to approximately 1 mg/mL, as previously described.^54^ Briefly, the HMO solutions were deposited on a matrix-assisted laser desorption/ionization (MALDI) target plate as follows: 1 μL of HMO was spotted followed by 0.2 μL of 10 mM NaCl and 1 μL of DHB matrix (60 mg/mL in 50% methanol). The spots were allowed to air-dry and were analyzed in positive ion mode on a 15 T Fourier transform ion cyclotron resonance (FT-ICR) mass spectrometer (MS) (Bruker Solarix). Mass spectra were acquired in the positive ion mode from m/z 300 to 2500. Sodium ion adducts of HMOs were detected with a mass accuracy of >2 ppm.

### Initial processing of infant gut metagenomic samples

Raw metagenomic reads from consenting individuals in the IGRAM cohort^50-52^ were processed using MetaPhlAn 4^55^ to create a merged taxonomic relative abundance table. Samples were included if indicated as breastfed only or formula-fed by mother. The sample sizes were as follows: breastfed, 1 month = 62; formula-fed 1 month = 93; breastfed, 4 months = 34; formula-fed, 4 months = 137.

### Gut metagenomic sialidase-encoding species database creation

Three publicly available gut metagenomic protein catalogs were obtained (MPA4 marker gene database, UHGP-100, and CMMG representative genomes).^55-57^ The MetaPhlAn 4 marker gene database was first converted from nucleic acid to protein sequences using Prodigal (v2.6.3).^58^ Enzyme structures and related literature were analyzed to create a putative structural rubric for sialidase enzymatic function as described previously.^37-39^ ^,^ ^59^ This structural rubric was employed to identify sialidase orthologs in all 3 databases. Resulting protein IDs were merged with their provided taxonomic annotations to create an integrated database of microbial species known to encode sialidases.

### Analysis of infant gut metagenomic sialidase-encoding species

The IGRAM species-level MetaPhlAn results were then filtered to only the species detected in the created sialidase database. These results were summed to generate percent abundance values for total sialidase-encoding species in each sample, as described for azoreductases by Simpson et al.^38^ Results were also summed by taxonomic level, yielding sialidase-encoding taxa abundance stratified by phyla, class, order, family, and genus. Linear mixed effects models were used to compare sialidase taxa abundance between feeding type and timepoints in infant gut metagenome samples. Sialidase taxa abundance was predicted as a function of the single fixed effect “specific feeding group” (feeding group combined with timepoint) as well as a random effect for the individual ID to account for repeated measurements from a single subject. *P*-values were obtained using the Kenward-Roger method.

### Statistics and analysis

Area under the curve was calculated for each growth curve using GraphPad Prism (v.11.0.0). Then, one-way analysis of variance (ANOVA) with Tukey's multiple comparisons test was performed using GraphPad Prism to determine statistical significance among different carbohydrate sources for each individual bacterium.

One-way ANOVA with Tukey's multiple comparisons test was performed using GraphPad Prism for all specific activity data. Specific activities of each sialidase were compared for 4MU-Neu5Ac and each individual pure HMO. Additionally, substrate specificity was determined by comparing the specific activity of each sialidase with each individual HMO.

Specific activity of mutated sialidases with 4MU-Neu5Ac, 3’SL, and 6’SL compared to their wild-type counterparts was analyzed for statistical significance using an unpaired t-test with Welch’s correction in GraphPad Prism.

## Results

### Differential growth on sialylated HMOs

To initially investigate the ability of human gut microbes to utilize sialylated HMOs as sole carbon sources, we anaerobically cultured *Bacteroides fragilis*, *Bacteroides thetaiotaomicron*, *Parabacteroides merdae,* and *Bifidobacterium longum* subsp. *infantis* with glucose, 6’SL, 3’SL, and FSA. While all grew on glucose, notable differences were observed with sialylated substrates, which were employed at 1% w/v in YCFA media for all taxa except *Bifidobacterium longum* subsp. *infantis* for which we used 2% w/v in MRS media^60^ ^,^ ^61^ (Figures 1B, C, and D, S1, S2). *B. fragilis* utilized both 6’SL and 3’SL with equal efficiency, reaching saturation in under 20 hours, but failed to grow on FSA (Figures 1B, S1A). By contrast, *B. thetaiotaomicron*, which also failed to utilize FSA, reached saturation in under 30 hours with glucose but showed only minimal growth after 40 hours with 6’SL and 3’SL (Figures 1C, S1B). Furthermore, *P. merdae* grew best on glucose, exhibited biphasic responses with 6’SL and 3’SL, and even showed moderate growth on FSA alone that was similar to the growth on 3’SL (Figures 1D, S1C). *Bifidobacterium longum* subsp. *infantis* showed only moderate growth on 3’SL and no growth on 6’SL or FSA even at the higher 2% w/v carbon source concentrations (Figure S2). Thus, these human gut microbial taxa demonstrate distinct abilities to utilize sialylated HMOs found in breastmilk as sole carbon sources.

### Identification of microbial sialidases in the adult and infant microbiome

To investigate the metabolic capabilities of diverse microbial sialidases, we utilized a structure-based rubric to define key sialidase features and identify unique sialidase sequences. We selected seven representative sialidase structures as reference sequences from the Protein Data Bank: *B. thetaiotaomicron* (4BBW),^29^ *Streptococcus pneumoniae* (5F9T),^41^ *Clostridium perfringens* (2BF6),^42^ *Alistipes sp.* (6MNJ),^43^ *Micromonospora virifaciens* (1EUR),^44^ *Cutibacterium acnes* (7LBU),^45^ and *Ruminococcus gnavus* (4X47) (Figure S3).^46^ All belong to the GH33 family with central 6-bladed *β*-propeller folds, while *B. thetaiotaomicron*, *S. pneumoniae*, and *Alistipes sp.* contain additional *N*-terminal carbohydrate-binding modules. We further selected five completely conserved active site residues required for sialidase function: a glutamate and tyrosine that are directly involved in the catalytic mechanism, along with an arginine triad that contacts sialic acid to orient substrates in the active site (Figure 2A).^21^ ^,^ ^62^

We then screened the IGC for microbial sialidases using this defined rubric. The IGC is a metagenomic database of sequenced fecal samples from 1267 healthy adult donor samples, resulting in 9,879,896 protein-encoding genes to query.^47^ We first identified protein sequences that showed at least 25% sequence identity to one of the seven representative sialidase structures that were selected (Figure 2B). Second, only those sequences that contained all five of the conserved active site residues were retained. Finally, the resultant set was condensed to combine sequences with >95% identity to produce a final list of unique proteins. Using this approach, we identified 351 unique microbial sialidases from the IGC, to which we then assigned taxonomy via BLASTP. An SSN colored by microbial phyla shows that sialidases are encoded primarily by Bacteroidota, with additional contributions from Bacillota, Verrucomicrobiota, and Actinomycetota (Figure 2C). Furthermore, one major cluster with 139 sequences was observed, along with 15 minor clusters containing 4 to 29 sequences that often grouped according to phylum. Based on this SSN, we selected 11 sequences from 9 major clusters, including sequences from all 4 major phyla, for recombinant protein expression, purification, and biochemical characterization: *B. thetaiotaomicron* (*Bt2*), *Akkermansia muciniphila* (*Am9*), *B. fragilis* (*Bf4*), *Alistipes dispar* (*Ad*), *Prevotella timonensis* (*Pt2*), *Ruminococcus lactaris* (*Rl*), *Bacteroides caccae* (*Bc6*), *P. merdae* (*Pm5*), *Dorea longicatena* (*Dl2*), and *Bifidobacterium longum* subsp. *infantis* (*Bli1* and *Bli2*).

To confirm that the chosen sequences were also relevant in the infant gut microbiome, we reemployed our stepwise structure-based rubric to identify microbial sialidases in the IGRAM dataset. IGRAM was a longitudinal birth cohort study from which metagenomic data were available for 153 healthy, full-term infants without antibiotic exposure, with samples collected from 4 d to 24 months after birth.^50^ ^,^ ^51^ We identified 214 unique sialidase sequences from IGRAM. The resultant SSN, colored according to phylum, reveals a similar pattern to that observed with adult IGC unique sialidases, with one central cluster and a set of smaller clusters grouped by microbial phylum (Figure 2D). We then determined which protein sequences from IGRAM most closely resembled those selected for *in vitro* analysis from the adult IGC database. We found that 10 of the 11 sequences from the IGC had an IGRAM counterpart with at least 80% sequence identity, and that 7 had >95% identity. The only exception was *Bc6*, which is a singleton with unclear taxonomy in the IGRAM SSN that shared only 32% identity with the corresponding Bacteroidota protein from the IGC. Thus, the infant and adult microbiomes encode a similar slate of gut microbial sialidase proteins and our set of proteins selected for characterization reflects the major taxonomic and SSN clusters present in both the IGC and IGRAM databases.

### Sialidase activity with human milk oligosaccharides

The 11 sialidase proteins were expressed and purified to homogeneity. In the cases of *Rl* and *Pt2*, we excluded from our constructs the additional N- and C-terminal domains present in these enzymes, and instead expressed only their central GH33 catalytic domains, regions analogous to those of the other 9 proteins purified. To examine general sialidase activity, we initially employed the reporter substrate 4-methylumbelliferyl-*α*-D-*N*-acetylneuraminic acid (4MU-Neu5Ac). We first conducted a pH screen to ensure that all active sialidases had >50% maximal activity at pH 6.5, a biologically relevant pH for the intestinal lumen (Figure S4).^63^ We then determined specific activity with 4MU-Neu5Ac for our panel of 11 sialidases at pH 6.5. A wide range of specific activities was observed with this reporter substrate. *Ad* showed a significantly higher specific activity, at 9.12 μM/s/μM, than the other enzymes, and was followed by a range of activities between 2 and 7 μM/s/μM for *Bt2, Rl*, *Bf4*, *Am9*, *Bli2*, and *Dl2*. *Bc6* and *Pm5* showed the lowest measurable specific activities under 0.5 μM/s/μM, while *Bli1* and *Pt2* exhibited no measurable activity (Figure 3A). Thus, 9 of the 11 proteins chosen showed sialidase activity with the reporter substrate 4MU-Neu5Ac, although with significantly different efficiencies (Table S3).

**Figure 3.**
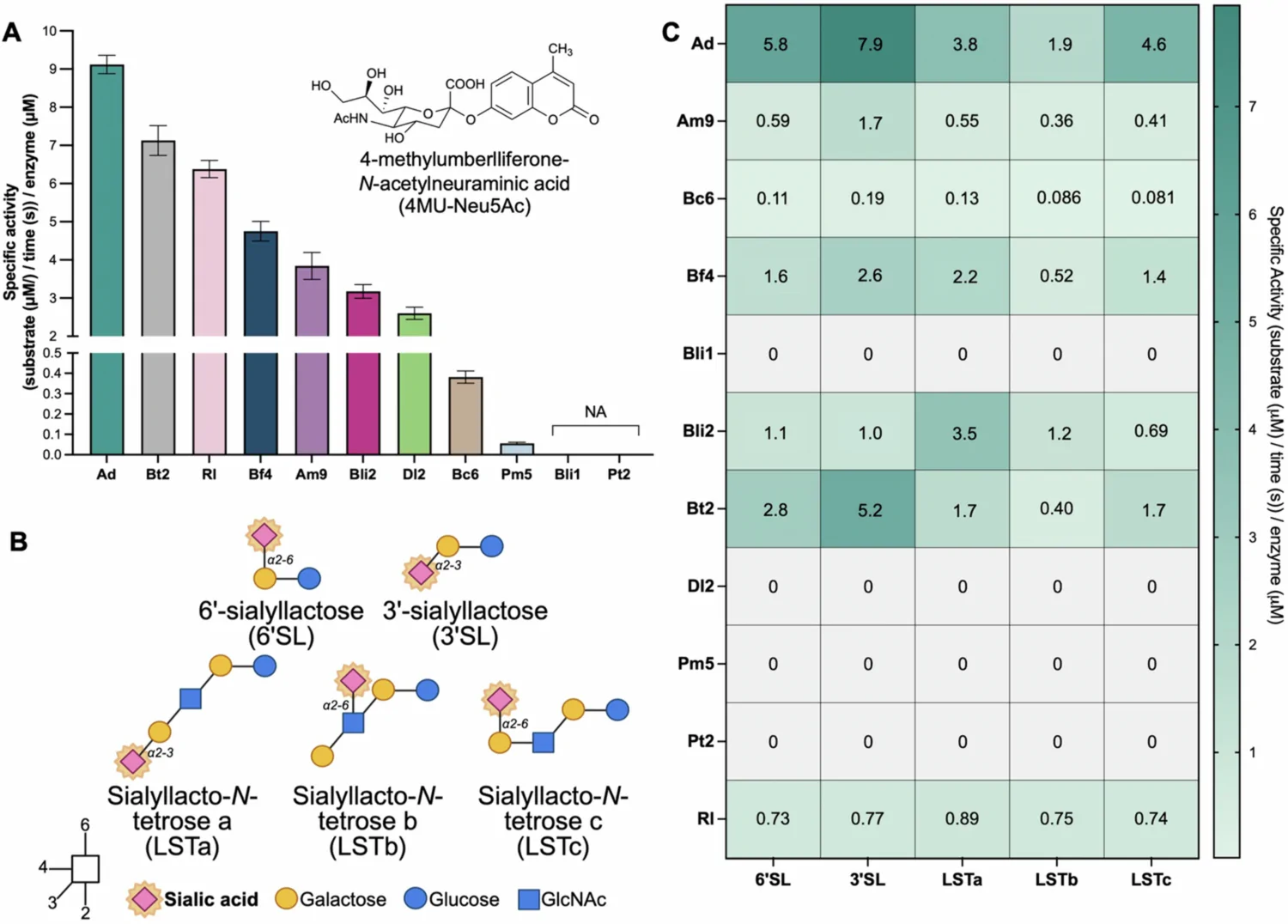
Purified microbial sialidases differentially process sialylated HMO substrates *in vitro*. (A) Specific activities of eleven purified microbial sialidases with reporter substrate 4MU-Neu5Ac. Error bars represent SEM of *N* = 3 biological replicates. (B) Schematic structures of monosialylated human milk oligosaccharides (HMOs) found in human breastmilk. (C) Specific activities of eleven purified bacterial sialidases with five monosialylated HMOs. *N* = 3 biological replicates.

We next examined a panel of monosialylated HMOs commonly found in breastmilk as substrates for our panel of purified sialidases. The HMOs chosen sample both α2-3 and α2-6 linkages, as well as different substrate lengths and sialic acid locations (Figure 3B). We determined specific activity with all 5 monosialylated HMOs for each of our 11 representative sialidases by measuring the release of sialic acid over time via LC-MS/MS (Figure 3C). We observed a wide range of specific activities. Again, *Ad* showed a significantly higher activity than the other enzymes with all HMOs, while *Bf4*, *Bli2*, and *Bt2* showed moderate activity, and *Am9*, *Bc6,* and *Rl* showed low activity (Figure S5, Tables S4–8). These results mirror those observed with the reporter substrate, described above. Similarly, *Bli1*, *Dl2*, *Pm5*, and *Pt2* exhibited no activity with all HMO substrates; these enzymes showed little to no activity with the reporter substrate 4MU-Neu5Ac as well. These data indicate that features beyond conserved fold and active site residues can influence the sialidase activities of putative enzymes selected from metagenomics databases.

For the active sialidases, we observed statistically different activities with individual enzymes and the panel of 5 HMOs, except for *Rl* that had consistent activity regardless of substrate (Figure S6, Tables S9–15). For the active sialidases with varying substrate specificities, we observed a general preference for α2-3 linkages; every active sialidase, except *Bli2 and Rl* (Tables S13 and S15), had a higher specific activity with 3’SL than 6’SL, which differ only in linkage to sialic acid (Figure 3B and C). We also observed a preference for shorter oligosaccharides, as evidenced by higher activity with 3’SL than sialyllacto-*N*-tetrose a (LSTa), both containing terminal α2-3 linkages, and 6’SL than sialyllacto-*N*-tetrose c (LSTc), both containing terminal α2-6 linkages. However, *Bli2* exhibited significantly higher specific activity with LSTa than 3’SL, and was the only differentially active sialidase to have a preferred substrate other than 3’SL. Finally, we found the lowest specific activity values with sialyllacto-*N*-tetrose b (LSTb) for the majority of the active sialidases. LSTb is unique in that the sialic acid is linked to an internal *N*-acetylglucosamine (GlcNAc) rather than a terminal galactose. Again, *Bli2* and *Rl* were exceptions, as they showed moderate activity with LSTb that were not statistically different than the activity with 3’SL and 6’SL. These results define key aspects of the selectivity of gut microbial sialidases for sialylated HMO substrates commonly found in human breast milk and allow for straightforward comparisons of sialidases from varying relevant taxa.

### Structural basis for differential gut microbial sialidase activities

To understand the molecular basis for distinctions in sialidase activities, we solved 3 microbial sialidase crystal structures: *Rl* and *Pm5*, along with *Ad* co-crystallized with FSA. The structure of *Bt2,* which has strong activity like *Ad* and *Rl*, was reported previously (PDB: 4BBW).^29^ Overall, these 4 protein structures span the spectrum of specific activities measured, as *Ad* shows high activity, *Rl and Bt2* show moderate activity, and *Pm5* shows trace to no activity with the substrates examined (Figure 3C, Tables S4–S8). We found that the 3 novel structures contained the expected central 6-bladed *β*-propeller fold and that *Rl* also contained an additional domain of 110 residues between two *β*-sheets (Figure 4A). The active site residues from the sialidase rubric were also conserved in all 4 structures, with minimal differences in side chain conformations (Figure 4B). In the co-crystal structure of *Ad* with sialic acid, the nucleophilic tyrosine is oriented towards carbon 2 of the sialic acid, which is the position where the nucleophilic attack would occur on an intact sialylated HMO. Sialic acid also forms polar contacts with the arginine triad, as well as an additional arginine and 2 aspartic acids (Figure S7). Furthermore, the sialic acid bound to *Ad* adopts the distorted conformation observed previously in a complex of 3’SL bound to a *trans*-sialidase (PDB: 1S0I).^26^

**Figure 4.**
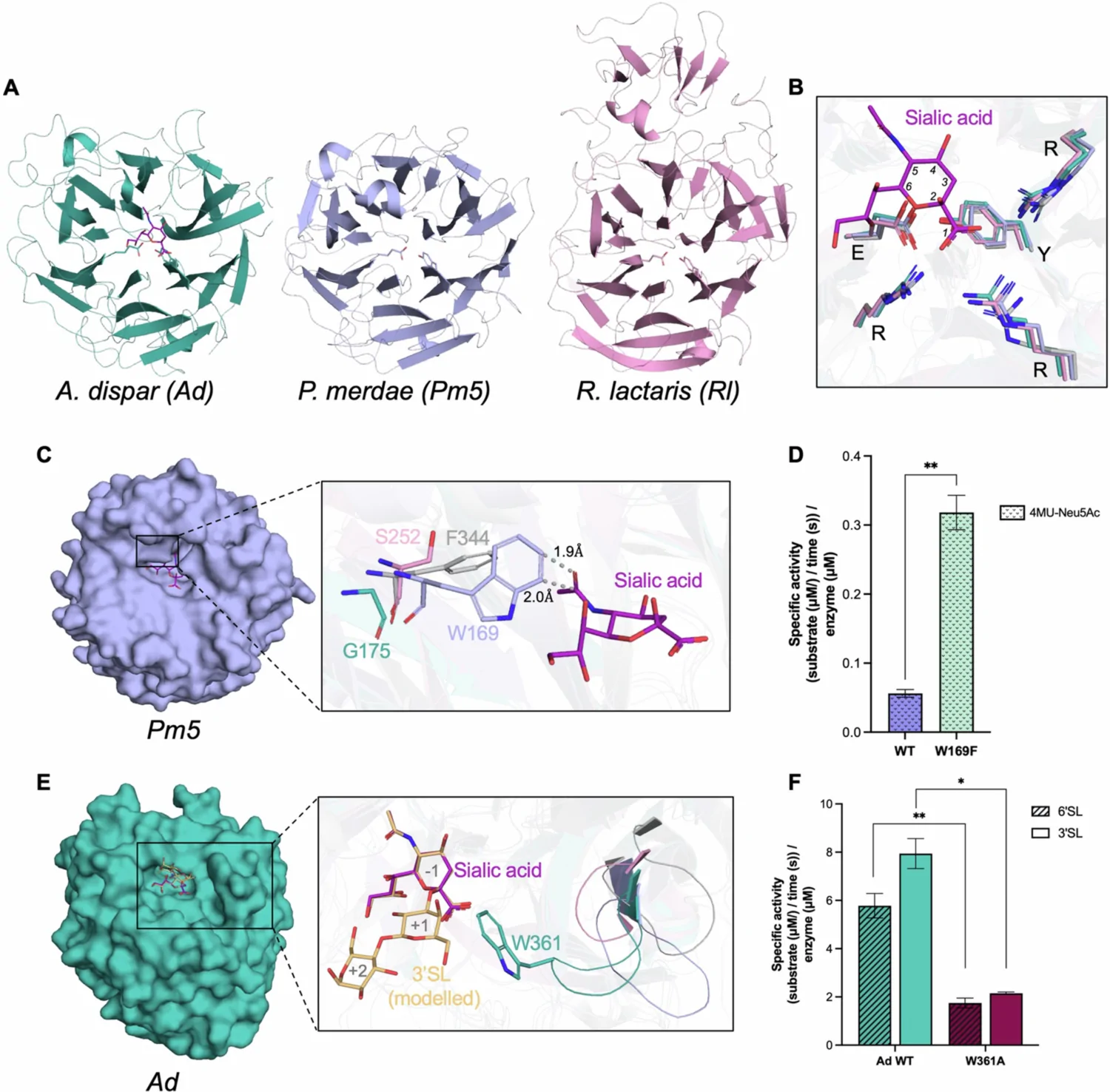
Structural insights into differential microbial sialidase activities. (A) Novel sialidase structures reported here from *A. dispar*, *P. merdae*, and *R. lactaris*. (B) Key conserved sialidase active site residues in all four analyzed structures (*Ad*: teal; *Pm5*: purple; *Rl*: pink; *Bt2* (4BBW): gray). (C) Surface representation of *Pm5.* Inset: Position of W169 in *Pm5* relative to the binding site for sialic acid. (D) Specific activity of wildtype *Pm* sialidase and W169F mutant with the reporter substrate 4MU-Neu5Ac. Error bars represent SEM of *N* = 3 biological replicates. The data were analyzed using an unpaired t-test with Welch’s correction (**P < 0.01). (E) Surface representation of 3’SL modeled in the *Ad* active site. Inset: Variable loops from *Ad* (teal), *Pm5* (purple), *Rl* (pink) and *Bt2* (gray) with the unique W361 from *Ad* shown. (F) Specific activities of wildtype *Ad* and W361A mutant *Ad* sialidases with 6’SL and 3’SL. Error bars represent SEM of *N* = 3 biological replicates. The data were analyzed using an unpaired t-test with Welch’s correction (*P < 0.05, **P < 0.01).

Given the conserved nature of the overall fold and key active site residues of these sialidases, we looked to adjacent regions to understand the functional differences we had observed. We found a unique tryptophan residue at position 169 in *Pm5*, which when overlayed with the co-crystal structure of *Ad* containing a glycine in this position, the tryptophan enters the sialic acid binding site (Figure 4C). The indole group of the tryptophan is only ~2 Å from the amide on carbon 5 of overlayed sialic acid moiety, which would create a steric clash and may explain why *Pm5* has minimal activity for sialylated substrates. By contrast, *Bt2* contains a phenylalanine at this position that does not appear to occupy the space required for sialic acid binding. To further investigate this, we used site-directed mutagenesis to replace W169 in *Pm5* with phenylalanine. We confirmed using circular dichroism and nanoDSF that *Pm5* W169F is folded, although it exhibits an increased melting temperature and evidence of enhanced secondary structure (Figure S8). We then assessed activity with 4MU-Neu5Ac, and 3’SL and 6’SL. With the reporter substrate 4MU-Neu5Ac, *Pm5* W169F showed a significant increase in specific activity Compared to wildtype *Pm5,* from 0.056 μM/s/μM for wildtype to 0.32 μM/s/μM for the mutant (Figure 4D). However, there was no quantifiable activity with 3’SL and 6’SL, indicating that alternative active site regions also contribute to the inability of *Pm5* to efficiently turnover sialylated HMOs.

We next modeled the binding of 3’SL into the active site of *Ad*, using the bound FSA as a guide, to identify other sidechains that may influence substrate binding. Doing so allowed us to identify a loop distinct in structure to *Ad* (Figure 4E). This loop is highly variable in both length (10–13 residues) and amino acid composition in the 4 structures examined here (Figure S9). The loop is unique in *Ad* because it contains a tryptophan (W361) not present in the other enzymes characterized. With 3’SL modeled in *Ad*, W361 is within ~4.2 Å of the galactose moiety of this substrate, while the glucose extends out of the active site. To examine the role of this residue in the efficient processing of 3’SL and 6’SL by *Ad*, we mutated it to alanine and performed activity assays with the reporter substrate 4MU-Neu5Ac (Figure S10), and 3’SL and 6’SL (Figure 4F). The mutation did not alter the overall structure of the variant enzyme as assessed by circular dichroism (Figure S11). Compared to wildtype *Ad, Ad* W361A showed significantly decreased activity with all tested substrates, down to levels that mirror observed activities of *Bt2* or *Bf4*, which lack a tryptophan in this loop (Figure 4E; Figure S9). Taken together, these results demonstrate the importance of active site-adjacent loops in influencing substrate utilization through contacts with both the sialic acid and non-sialic acid portions of the substrate.

### MALDI-FT-ICR MS/MS HMO profiling reveals donor Lewis blood group

It is well established that HMO expression patterns vary from donor to donor and depend on various factors, including the stage of lactation, infant needs, and maternal health and lifestyle, among others.^10^ ^,^ ^64^ However, the main determinant of overall HMO composition is the mother’s secretor and Lewis blood group status, which can be identified through mass spectrometry analysis of the neutral/fucosylated HMO fraction. Following the method developed by Kunz and coworkers,^65^ we examined fragmentation patterns in the spectra of the isolated neutral/fucosylated HMOs (Figure S12). The presence of the 511 *m/z* fragment indicates that this donor is a secretor-type, either Le(a-b+) or Le(a-b-). Secretor status reflects FUT2 activity, which enables the synthesis of α1-2 fucosylated HMOs. To distinguish between these two Lewis types, we next analyzed the fragmentation ions at *m/z* 730 and 876. Because the peak at *m/z* 730 was significantly lower in intensity than the peak at *m/z* 876, we determined that this donor is Le(a-b+). Mothers in this blood group can produce milk with α1-2, α1-3, and α1-4 fucosylated HMOs.^66^

### Abundant sialylated HMOs identified by MS

Secretor and Lewis status also indirectly influence the composition of sialylated HMOs by altering substrate availability for sialyltransferases. However, several acidic HMOs are commonly found across all blood groups, including 3’SL, 6’SL, LSTa, LSTb, LSTc, and disialyllacto-*N*-tetraose (DSLNT) because their structures do not depend on fucosylation patterns (Figure 5A).^10^ ^,^ ^13^ ^,^ ^67^ Both 3’SL and 6’SL consist of lactose with an α2-3-linked or α2-6-linked sialic acid, respectively, on the galactose residue. Lacto-*N*-tetraose is the core structure of LSTa, LSTb, and DSLNT. LSTa bears a terminal α2-3-linked sialic acid while LSTb contains an α2-6-linked sialic acid on the GlcNAc residue. DSLNT contains both the terminal α2-3 sialic acid and the α2-6-linked sialylation on GlcNAc. LSTc, by contrast, has a lacto-*N*-neotetraose core with a terminal 2-6-linked sialic acid. Each of these commonly observed acidic HMOs was identified in the milk of the donor used herein (Figure S12).

**Figure 5.**
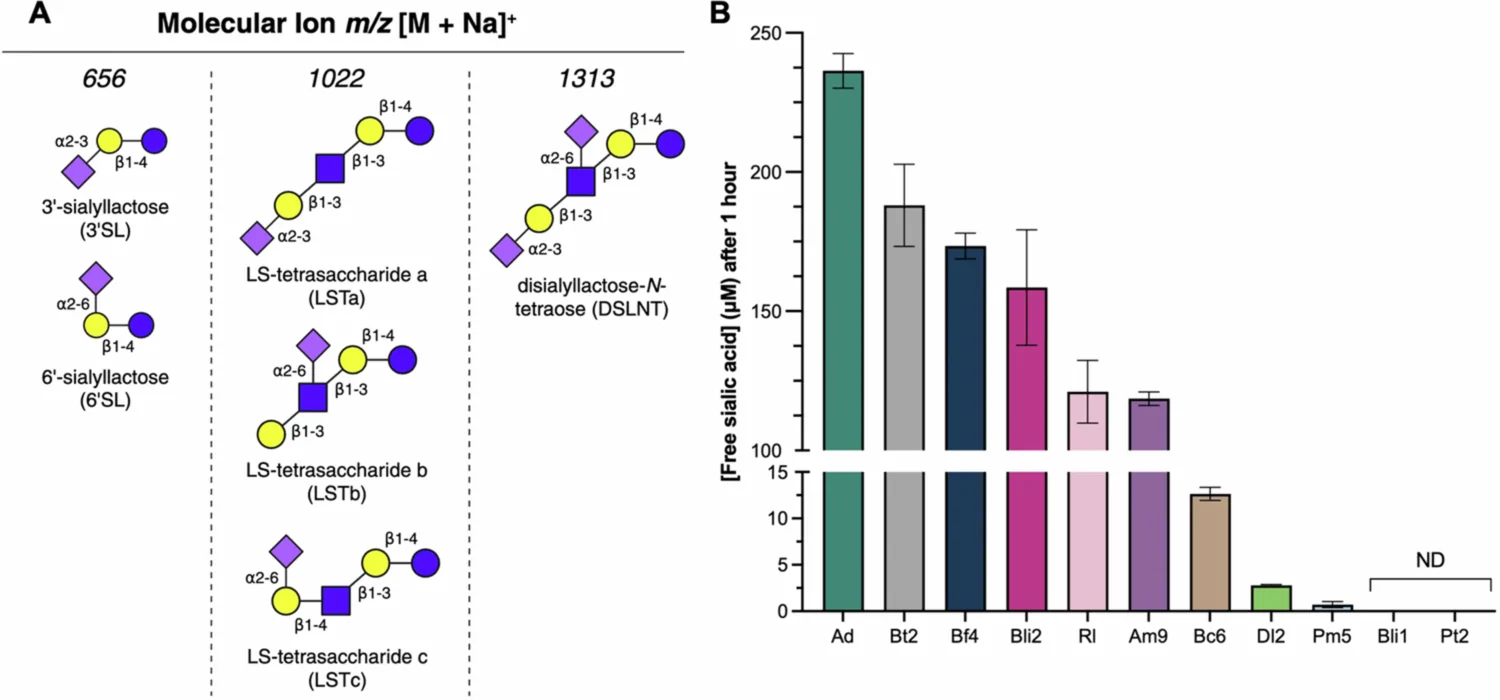
Sialylated HMOs isolated from a human breastmilk sample are processed by microbial sialidases. (A) Schematic structures and *m/z* values for purified sialylated HMOs in the human breast milk sample examined. (B) Free sialic acid concentrations produced by each enzyme after 1 hour incubations with 0.5 mg/mL of the isolated sialylated HMOs mixture shown in panel A. ND, not detected. Error bars represent SEM of *N* = 3 biological replicates.

### Sialidase activity with HMOs from donor breastmilk

Using the isolated and fully characterized sialylated HMO mixture from the breastmilk donor sample, we examined the ability of each of the purified sialidases to produce FSA (Figure 5B). Concentrations of FSA after 1-hour incubations with constant concentrations of purified sialidase were measured to compare general sialidase activity with this mixture. Like the activity trends observed with the individual sialylated HMOs, we see distinct capabilities to process the isolated mixture. Again, *Ad* was the most efficient sialidase in the panel, producing 236.4 μM FSA in the 1-hour timeframe. Next, *Bt2*, *Bf4*, and *Bli2* exhibited similar sialidase capabilities, producing 158.5 μM up to 188.0 μM FSA. *Rl* and *Am9* showed moderate activity, and *Bc6* had low activity, which again follows the general trend observed with the calculated specific activities. Interestingly, there were low, but still detectable, amounts of FSA produced by *Dl2* and *Pm5*. We were unable to determine reaction rates for these enzymes in our specific activity panel, but simply quantifying FSA in this assay allows us to make more nuanced observations and corroborates the activity observed with 4MU-Neu5Ac. Finally, both *Bli1* and *Pt2* released no detectible FSA, which again is consistent with both the HMO specific activity panel as well as the 4MU-Neu5Ac specific activity.

### Infant sialidase-encoding microbial metagenomic differences by feeding method

Finally, we examined whole genome metagenomics collected from infant stool samples in the IGRAM study^50^ ^,^ ^51^ to investigate whether feeding method influenced sialidase-encoding bacterial taxa in the infant gut. We focused on data obtained at 1 and 4 months of age and from exclusively breastfed (mother reported feeding breastmilk and not formula) or formula-fed infants. Both timepoints occur before the typical introduction of solid foods at 6 months. At 1 month, breastfed infants had higher abundances of sialidase-encoding bacterial species than their formula-fed counterparts (Figure 6A). By 4 months of age, this difference was lost, as formula-fed infants showed a significant increase in sialidase-encoding species between 1 and 4 months of age. The breastfed microbiomes remained largely unchanged from 1 to 4 months, while the formula-fed microbiomes exhibited more fluctuation in taxonomic composition. Thus, during early colonization, breastfed babies obtained significantly more sialidase-encoding taxa at 1 month than formula-fed infants.

**Figure 6.**
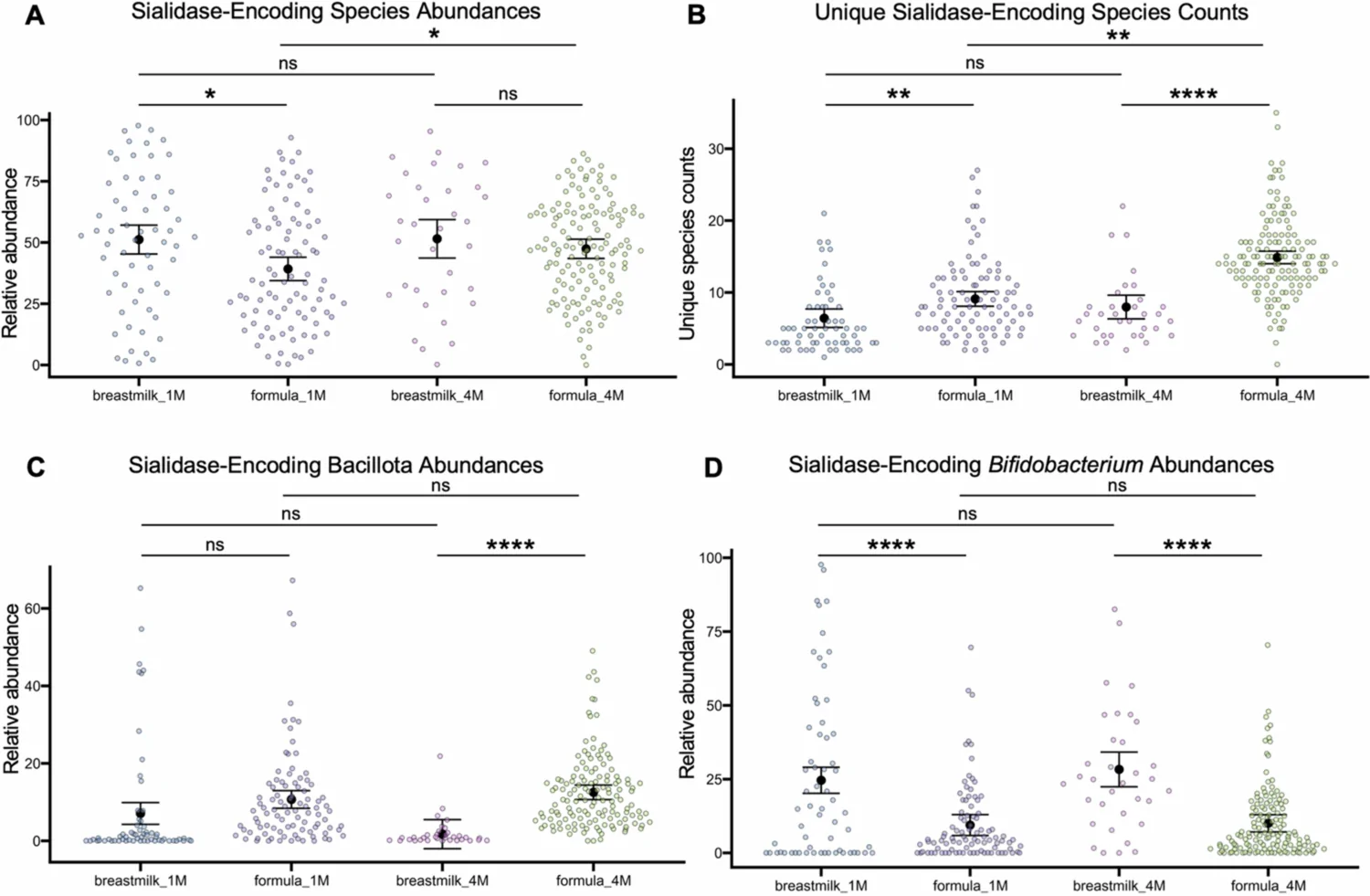
Breastfed and formula-fed infants exhibit distinct sialidase-encoding taxa in early life. (A) Sialidase-encoding species relative abundance by feeding method and age. (B) Unique species counts of sialidase-encoding species by feeding method and age. (C) Relative abundances of sialidase-encoding Bacillota by feeding method and age. (D) Relative abundances of sialidase-encoding *Bifidobacterium* by feeding method and age. All data analyzed using the Kenward-Roger method (*P < 0.05, **P < 0.01, ***P < 0.001, ****P < 0.0001, ns = not significant).

We next examined the number (counts) of unique sialidase-encoding species present in samples, which provides a perspective of the diversity of the bacteria capable of producing sialidases. We found that formula-fed infants had higher numbers of unique sialidase-encoding species at 1 month and 4 months than breastfed infants (Figure 6B). Furthermore, formula-fed infants gained significantly more sialidase-encoding species between 1 and 4 months, while breastfed babies did not. Thus, while breastfed infants showed a higher relative abundance of taxa encoding sialidases at 1 month (i.e., more of the taxa present at that time encoded one or more sialidase genes), formula-fed infants showed higher numbers of sialidase-encoding taxa present and the unique capability of changing these taxa over the first 4 months of life.

Given the unique species profiles in breastfed and formula-fed infants at 1 and 4 months of age, we next sought to determine the specific taxa driving these differences. We found that formula-fed infants showed a trend toward a higher abundance of sialidase-encoding Bacillota at 1 month, and a statistically significantly higher abundance of Bacillota at 4 months (Figure 6C). By contrast, some breastfed samples showed relatively high Bacillota abundance at 1 month, but by 4 months, the relative abundance of sialidase-encoding Bacillota taxa is near zero for breastfed babies. These results indicate that formula better supports the growth of sialidase-encoding Bacillota than breastmilk during these stages of early life.

Since formula-fed infants show an abundance of sialidase-encoding Bacillota, we lastly sought to determine which sialidase-encoding taxa were differentially colonizing breastfed infants. We found that the abundance of sialidase-encoding microbes from the *Bifidobacterium* genus was significantly higher in breastfed infants at both 1 month and 4 months of age (Figure 6D). In addition, the *Bifidobacterium* abundance levels did not change between the groups between 1 and 4 months. These results indicate that breastfeeding supports the stable colonization of higher levels of sialidase-encoding *Bifidobacterium* taxa, which are known to be beneficial, compared to formula feeding. Sialidase activity may play a crucial role in this process because several HMOs contain sialylated moieties, while formula is known to lack sialylated compounds.^15^ Taken together, these results indicate that feeding type plays a significant role in the colonization of microbes encoding sialidase enzymes prior to the introduction of solid food.

## Discussion

We find that gut microbes cultured anaerobically differentially utilize sialylated substrates for growth. Previous studies had examined the abilities of different strains of *Bifidobacterium* and *Bacteroides* to grow on sialylated and fucosylated HMOs.^68-72^ For *Bifidobacterium,* the type strain of *B. longum* subsp. *infantis*has been examined as a representative of the infant gut microbiome, although its growth on different carbon sources has not been consistent. Some studies report high growth on 3’SL^69^ and 6’SL,^60^ ^,^ ^61^ others show moderate growth on 3’SL,^60^ ^,^ ^70^ 6’SL,^70^ and FSA,^68^ and some report low to no growth on 6’SL^69^ and FSA.^70^ In our study, *B. longum* subsp. *infantis* showed moderate growth on 3’SL, but no growth on 6’SL and FSA (Figure S2). Beyond *Bifidobacterium*, *B. thetaiotaomicron* showed limited abilities to grow on 3’SL, 6’SL or FSA, *B. fragilis* grew robustly on 3’SL and 6’SL but weakly on FSA, and *P. merdae* appears to use all carbon sources reasonably well for growth (Figure 1). Thus, taken together with previous reports, these data advance our understanding of how individual gut microbial taxa employ sialylated compounds for growth in anaerobic conditions.

Our metagenomic analyzes of IGRAM indicate that specific bacterial species that produce sialidases appear better equipped to grow with sialylated HMO-rich diets. This is evidenced by the higher abundances of sialidase-encoding species in the breastfed cohort at 1 month after birth (Figure 6A). Sialylated HMOs are most abundant in breastmilk during early lactation and decrease as the infant grows.^14^ ^,^ ^73^ Microbes producing sialidases are thus likely to be able to colonize such a sialylated HMO-rich environment, and continue to thrive in future months, even as sialylated HMO concentrations begin to decrease. Alternatively, formula, which contains 100-fold less sialylated compounds, would likely support other taxa and would take longer for sialidase-encoding microbes to colonize, as we find here (Figure 6A). However, we observe an increase in sialidase-encoding species in the formula-fed group at 4 months, making their abundances comparable with the breastfed group at this timepoint. This is consistent with previous studies that suggested breastfed infants have less diverse microbiomes, and are also less likely to change over time prior to the introduction of solid foods.^3^ ^,^ ^5^ This lack of elaboration over time for breastfed gut microbiomes is thought to be driven by the consistent predominance of *Bifidobacterium* in these environments, which was also observed in our analysis (Figure 6D).^3^ Additionally, we show that at 4 months, sialidase-encoding Bacillota are more abundant in formula-fed infants than breastfed infants (Figure 6C). Conversely, in breastfed infants, *Bifidobacterium* accounts for a significantly higher proportion of the sialidase-encoding bacteria at both 1 and 4 months (Figure 6D), even nearing 100% relative abundance in some samples, suggesting that the composition of sialylated molecules in breastmilk better supports the growth of *Bifidobacterium*.

Rationale for these functional differences was obtained using novel structural information. Our site-directed mutagenesis of W169 in *Pm5* provides unique insights into the structural components driving sialidase enzymatic efficiency. Upon mutating the tryptophan to a phenylalanine, we observe increased specific activity with the reporter substrate 4MU-Neu5Ac, but no quantifiable specific activity with either 3’SL or 6’SL (Figure 4D). This indicates that the addition of a smaller amino acid at this position facilitates sialic acid binding in the active site, evidenced by the increased activity with the smaller reporter substrate. However, it also indicates that sites other than the direct sialic acid binding site are important for accommodation of larger substrates and overall HMO processing ability. Indeed, our studies probing the functionality of *Ad* demonstrates the importance of regions outside of the sialic acid binding site. The mutation to residue 361 in *Ad* resulted in a decreased, but not eliminated, activity with 3’SL and 6’SL despite having no direct interactions with the sialic acid portion of these molecules. The loop region identified to promote activity in *Ad* is the same area that defines intramolecular-*trans* (IT) sialidases. Previous studies have shown that IT-sialidases have stringent substrate preferences for α2-3 linkages because of a tryptophan and tyrosine forming a conserved hydrophobic stack.^20^ ^,^ ^46^ Additionally, the structure of the IT-sialidase from *R. gnavus* (4X47),^46^ which was used in our sialidase rubric creation, contains a much longer loop in this region (Figure S13). However, we show that *Ad* contains a medium-length, tryptophan-containing loop in this same region, yet has the highest specific activity with both α2-3 and α2-6 linkages. We further find that this tryptophan is essential for providing the increased functionality, as evidenced by our mutagenesis studies (Figure 4F). Thus, within this region of gut microbial sialidases, both loop length and amino acid composition appear critical to optimize sialidase activity with HMO substrates. Taken together, these findings provide a deeper understanding of the roles that select active site residues and regions play in overall sialidase function.

Based on our data, we suggest that 3’SL is an optimal candidate for formula supplementation to promote infant gut microbiome health. All the active sialidases in our panel had notable turnover rates with 3’SL, and 3’SL was also the preferred substrate for the majority of those enzymes (Figure 3C). Additionally, all four of the microbes we cultured were able to use 3’SL as a sole carbon source, albeit with varying efficiencies (Figures 1, S2). Furthermore, three of the four microbes we cultured were unable to grow using FSA. In the gut, the sialic acid could instead be absorbed by the infant intestine to aid in neurodevelopment and exert other benefits of this sugar.^12^ ^,^ ^18^ ^,^ ^23^ 3’SL accounts for ~25% of all monosialylated HMOs in breastmilk, meaning that 3’SL is one of the major sources of sialylated compounds in breastfed infants.^73^ Therefore, it is likely that 3’SL influences the differences in sialidase-encoding microbes observed in IGRAM, and its supplementation in formula could shape the gut microbiome and enhance the development of infants who are not able to consume significant amounts of breastmilk.

Previous studies have outlined the differences between breastfed and formula-fed infants in regards to health outcomes and gut microbiome composition, and have suggested that HMOs are major contributors to these distinctions.^3^ ^,^ ^4^ ^,^ ^7^ ^,^ ^74^ However, the molecular mechanism by which this occurs has not been fully elucidated. We suggest that the presence of specific microbial sialidases is a key mechanism by which certain bacteria flourish in the uniquely curated intestinal environment of breastfed infants. Breastmilk and formula contain markedly different oligosaccharide compositions, specifically those that are sialylated. Mature breastmilk contains 2–3 g/L sialylated HMOs, whereas comparable formulas only have 0.05–0.1 g/L.^15^ In the breastmilk donor sample that was subsequently used to assess sialidase functionality, we found that the sample contained all of the commonly sialylated HMOs that are abundant in breastmilk (Figure S12). We observed diverse abilities of relevant sialidases to remove sialic acid from these sialylated HMOs in biologically relevant ratios *in vitro* (Figure 5B). This study confirms the ability of microbial sialidases to process structurally complex mixtures of HMOs in biologically relevant concentrations and relative abundances.

In summary, we have shown that distinct sialidases differentially process sialylated HMOs, and we have pinpointed key structural features involved. We suggest that sialidases play a critical role in shaping the infant gut microbiome. Using the key groundwork established here, future work will aim to pinpoint specific sialidases in these beneficial taxa contributing to the observed microbiome differences. Additional future studies investigating the role of microbial cross-feeding will be essential in understanding the dynamic nature of the infant microbiome, and the fate of FSA in the infant gut. Furthermore, additional studies examining other glycoside hydrolase enzymes will be necessary to fully understand the true scope of the interactions between gut microbes and HMOs in breastmilk. Nevertheless, our current findings provide unique insights into the establishment of the infant gut microbiome and suggest future ways to improve the composition of infant formula.

## Supplementary Material

Supplementary Material Supplementary_Figure_Caption.docx

Supplementary Material Plisko_etal_FigureS1_Revised_2.jpg

Supplementary Material Plisko_etal_FigureS2_Revised_2.jpg

Supplementary Material Plisko_etal_FigureS3_Revised_2.jpg

Supplementary Material Plisko_etal_FigureS4_Revised_2.jpg

Supplementary Material Plisko_etal_FigureS5_Revised_2.jpg

Supplementary Material Plisko_etal_FigureS6_Revised_2.jpg

Supplementary Material Plisko_etal_FigureS7_Revised_2.jpg

Supplementary Material Plisko_etal_FigureS8_Revised_2.jpg

Supplementary Material Plisko_etal_FigureS9_Revised_2.jpg

Supplementary Material Plisko_etal_FigureS10_Revised_2.jpg

Supplementary Material Plisko_etal_FigureS11_Revised_2.jpg

Supplementary Material Plisko_etal_FigureS12_Revised_2.jpg

Supplementary Material Plisko_etal_FigureS13_Revised_2.jpg

## Data Availability

The datasets supporting this work are included within the article and supplemental files. All coordinate files for reported crystal structures are available through the RCSB Protein Data Bank (https://www.rcsb.org/) under accession codes 10EI, 10EM, and 10EW.
