# Supplementary material for: Human milk oligosaccharides are distinctly processed by early life gut microbial sialidases
Source: Gut Microbes. 2026 Oct 29;18(1):2748788. doi: 10.1080/19490976.2026.2748788 (PMC13733203; doi:10.1080/19490976.2026.2748788)
Supplement: Supplementary file 1 [file KGMI_A_2748788_SM1807.docx]

**Figure S1.** Calculated area under the curve for bacterial growth assays. Area under the curve was calculated for measured growth curves on glucose, 6’SL, 3’SL, and FSA for *B. fragilis* (**A**), *B. thetaiotaomicron* (**B**), and *P. merdae* (**C**). Statistical significance was determined using one-way ANOVA with Tukey’s multiple comparisons test method (**p<0.05, **p<0.01, ***p<0.001, ****p<0.0001)*.

**Figure S2.** Anaerobic culturing of *B. longum* subsp. *infantis* with sialylated carbon sources. (A) Growth curves of B. longum subsp. infantis with 2% w/v of the following as sole carbon sources: glucose, lactose, 6’SL, 3’SL, and FSA. (B) Calculated area under the curve for *B. longum* subsp. *infantis* growth curves. Statistical significance was determined using one-way ANOVA with Tukey’s multiple comparisons test method (*****p<0.0001)*.

**Figure S3.** Multiple sequence alignment of reference sialidase sequences. Nucleophilic tyrosine and structurally adjacent glutamic acid residues are highlighted in cyan, and the arginine triad is highlighted in magenta*.*

**Figure S4.** pH screen of purified sialidase activities from 3.0-8.0. Relative [FSA] was determined using the reporter substrate 4MU-Neu5Ac by calculating the concentration of free sialic acid after 25 minutes and dividing by the highest concentration of FSA produced in that replicate. N=3 biological replicates.

**Figure S5.** Multiple comparison of specific activity with individual sialylated HMOs. The specific activity of all 11 purified sialidases was statistically compared for 6’SL (**A**), 3’SL (**B**), LSTa (**C**), LSTb (**D**), and LSTc (**E**). Each bar is labeled based on significant differences from one-way ANOVA with Tukey’s multiple comparisons test. Bars with multiple letter labels indicate lack of significant differences with multiple groups. Error bars represent SEM of N=3 biological replicates.

**Figure S6.** Multiple comparison of specific activity of each active sialidase. The specific activity with all 5 pure sialyalated HMOs was statistically compared for *Ad* (**A**), *Am9* (**B**), *Bf4* (**C**), *Bli2* (**D**), *Bc6* (**E**), *Bt2* (**F**), and *Rl* (**G**). Each bar is labeled based on significant differences from one-way ANOVA with Tukey’s multiple comparisons test. Bars with multiple letter labels indicate lack of significant differences with multiple groups. Error bars represent SEM of N=3 biological replicates.

**Figure S7.** Polar contacts to free sialic acid in structure of *Ad* sialidase. All contacts were determined using PyMOL. Residues included in sialidase rubric are labeled in bold.

**Figure S8.** Examination of wildtype and W169F mutant forms of *Pm5* secondary structure. (**A**) Circular dichroism spectra at 25°C. Scans were corrected for background signal by subtracting out data from a blank scan. (**B**) Circular dichroism-monitored thermal denaturation profiles examined at 215 nm from 25-95°C. (**C**) NanoDSF thermogram of ratio of integrated fluorescence at 350nm/330nm collected from 25-95°C.

**Figure S9.** Multiple sequence alignment of the unique loop region in sialidases of known structure. Residues in the identified loop region are colored according to protein (*Rl*: pink; *Bt2*: gray; *Pm5*: purple; *Ad*: teal). The tryptophan examined by mutagenesis in *Ad* is highlighted in orange.

**Figure S10.** Specific activity of wildtype *Ad* sialidase and W361A mutant *Ad* sialidase with the reporter substrate 4MU-Neu5Ac. Error bars represent SEM of N=3 biological replicates. The data were analyzed using an unpaired t-test with Welch’s correction (****p<0.001).*

**Figure S11.** Examination of wildtype and W361A mutant forms of *Ad* by circular dichroism. (**A**) Circular dichroism spectra at 25°C. Scans were corrected for background signal by subtracting out data from a blank scan. (**B**) Circular dichroism-monitored thermal denaturation profiles examined at 215 nm from 25-95°C.

**Figure S12.** MALDI-FT-ICR-MS/MS spectra of (top) the neutral/fucosylated fraction and (bottom) the charged sialylated fraction across 0-2500 *m/z* a single donor obtained in the positive ion mode.

**Figure S13.** Comparison of active site loop regions *R. gnavus* IT-sialidase and *Ad* hydrolytic sialidase. (*Ad*: teal; *R. gnavus*: dark blue). The defining tyrosine/tryptophan hydrophobic stack in *R. gnavus*, and the tryptophan of interest in *Ad* are highlighted.
